# A Perlin Noise-Based Augmentation Strategy for Deep Learning with Small Data Samples of HRCT Images

**DOI:** 10.1038/s41598-018-36047-2

**Published:** 2018-12-06

**Authors:** Hyun-Jin Bae, Chang-Wook Kim, Namju Kim, BeomHee Park, Namkug Kim, Joon Beom Seo, Sang Min Lee

**Affiliations:** 1 Department of Convergence Medicine, University of Ulsan College of Medicine & Asan Medical Center, Seoul, Republic of Korea; 2 Kakao Brain Inc, Seoul, Republic of Korea; 3 Department of Radiology, University of Ulsan College of Medicine & Asan Medical Center, Seoul, Republic of Korea

## Abstract

Deep learning is now widely used as an efficient tool for medical image classification and segmentation. However, conventional machine learning techniques are still more accurate than deep learning when only a small dataset is available. In this study, we present a general data augmentation strategy using Perlin noise, applying it to pixel-by-pixel image classification and quantification of various kinds of image patterns of diffuse interstitial lung disease (DILD). Using retrospectively obtained high-resolution computed tomography (HRCT) images from 106 patients, 100 regions-of-interest (ROIs) for each of six classes of image patterns (normal, ground-glass opacity, reticular opacity, honeycombing, emphysema, and consolidation) were selected for deep learning classification by experienced thoracic radiologists. For extra-validation, the deep learning quantification of the six classification patterns was evaluated for 92 HRCT whole lung images for which hand-labeled segmentation masks created by two experienced radiologists were available. FusionNet, a convolutional neural network (CNN), was used for training, test, and extra-validation on classifications of DILD image patterns. The accuracy of FusionNet with data augmentation using Perlin noise (89.5%, 49.8%, and 55.0% for ROI-based classification and whole lung quantifications by two radiologists, respectively) was significantly higher than that with conventional data augmentation (82.1%, 45.7%, and 49.9%, respectively). This data augmentation strategy using Perlin noise could be widely applied to deep learning studies for image classification and segmentation, especially in cases with relatively small datasets.

## Introduction

Deep neural networks (i.e., deep learning systems) are becoming increasingly powerful tools for solving medical imaging problems such as medical image segmentation and classification^1,2^. Deep learning mimics the complex neural connections of human brains to automatically learn low- to high-level features of a given dataset, and to then identify the most significant features in the data^3^. Compared with classical machine learning techniques such as support vector machine (SVM) classifiers, deep learning-based methods provide more successful and reliable results in most clinical applications at the expense of more hardware resources^4^.

While deep learning is widespread in many research fields, the creation of the appropriate deep learning models for each application could be challenging due to several reasons. One of the most challenging issues is a paucity of human-labeled data and its expenses, especially in medical research. For supervised learning, such as is the case with convolutional neural networks (CNNs) or recurrent neural networks (RNNs), it is critical to have a large amount of high-quality human-labeled data, which are generally very expensive to produce. To overcome shortages in human-labeled data, a reliable and efficient data augmentation strategy is critical for deep learning.

Here, we present a novel Perlin noise-based data augmentation strategy for deep learning on medical images. Compared with classical noise-generation algorithms, Perlin noise provides random but natural-appearing patterns or textures, with little computational cost^5,6^. The algorithm for Perlin noise is simple, and can be applied in multiple dimensions, e.g., 1D, 2D, or 3D. Because of its characteristics, Perlin noise has been widely used in computer graphics to represent natural patterns such as fires or clouds^7^.

To validate the usefulness of our strategy, we performed deep learning experiments to automatically quantify complex image patterns of diffuse interstitial lung disease (DILD) on 2D images of high-resolution computed tomography (HRCT). DILD is a complex group of disorders affecting the lung parenchyma, which lead to respiratory failure if the cause is not removed or if therapy fails^8^. As HRCT can perform accurate and rapid image assessments of lung parenchyma non-invasively, HRCT has become an essential diagnostic imaging modality for investigating DILD^9,10^. However, there is considerable inter- and intra-observer variation in the interpretation of HRCT for diagnosis of DILD, with there being both a lack of standard criteria and the burden of reviewing a large amount of data^11^. In this regard, a computer-aided diagnosis (CAD) scheme that can reduce variations in clinical interpretations is important, especially for the quantification of DILD in HRCT images. Perlin noise could be used to make a theoretically infinite number of random mixtures of different DILD disease patterns from 2D HRCT images, which should lead to improved accuracy in the deep learning of DILD classifications in comparison with that in previous studies.

This paper is constructed as follows. In Section 2, we introduce the materials and methods in detail. We present the results of our experiments in Section 3 and discuss them in Section 4, and summarize and conclude the study in Section 5.

## Materials and Methods

### Subjects

**Figure 1:**
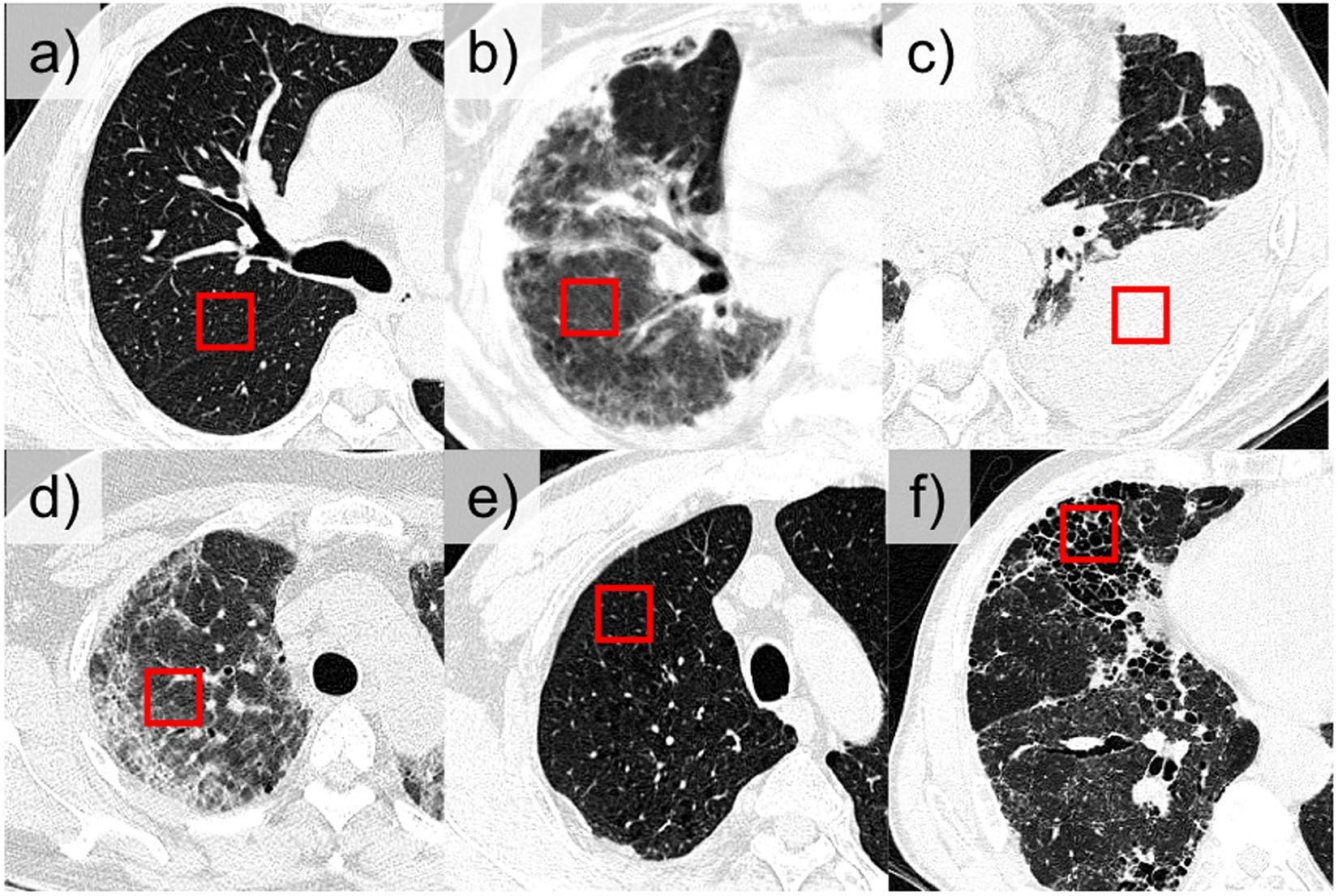
Examples of 2D HRCT lung parenchyma images with ROIs (red box) demonstrating (**a**) normal, (**b**) ground-glass opacity, (**c**) consolidation, (**d**) reticular opacity, (**e**) emphysema, and (**f**) honeycombing patterns.

HRCT images obtained on a Siemens CT scanner (Sensation 16, Siemens Medical Solutions, Forchheim, Germany) were selected for 106 patients, including 36 patients with usual interstitial pneumonia, 35 patients with cryptogenic organizing pneumonia, 16 patients with emphysema, 4 patients with pneumonia, 1 patient with acute interstitial pneumonia, and 14 subjects with no DILD. The typical HRCT parameters included 220 mAs and 120–140 kVp. Images were reconstructed with a 1 mm slice thickness at 10 mm intervals using a B70f enhancing reconstruction kernel. The typical voxel size of HRCT is 0.5–075 × 0.5–075 × 1 mm. The CT images were acquired with breath-holding at full inspiration following the radiographer’s instructions. Figure 1 shows an example of each class of DILD pattern in the 2D HRCT images from the subjects. The institutional review board for human investigations at Asan Medical Center (AMC) approved the study protocol with removal of all patient identifiers from the images, and waived the requirement for informed consent, in accordance with the retrospective design of this study.

**Figure 2:**
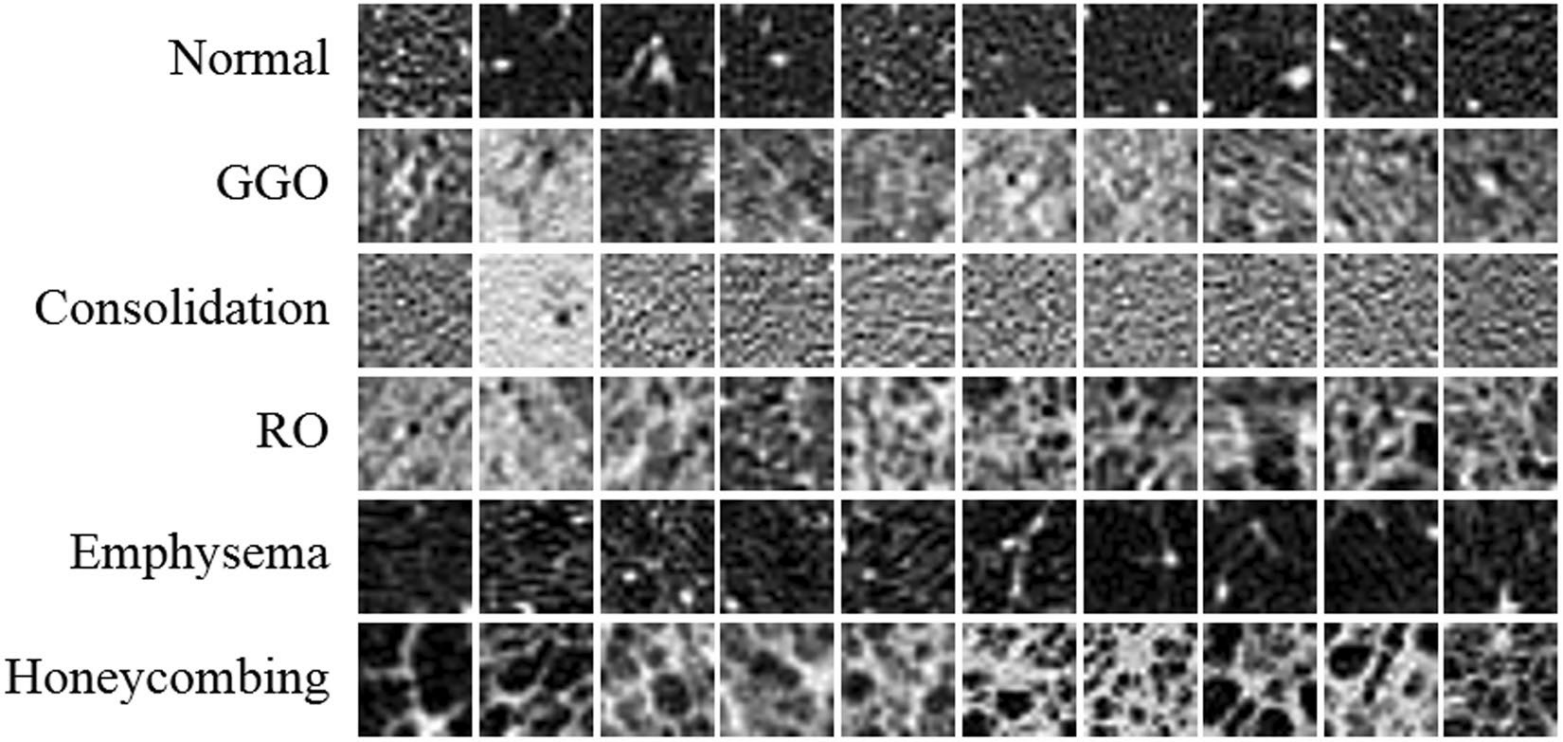
Examples of 10 ROIs (size of 20 × 20 pixel) of each class of DILD image patterns in HRCT.

As it was possible for regions to simultaneously demonstrate two or more patterns, making it difficult to achieve a consensus for even expert radiologists, two thoracic radiologists with more than 10 years of experience and working in consensus were asked to label 2D 20 × 20 pixel regions-of-interest (ROIs) on the HRCT images. The ROIs were classified into one of five DILD classes or a normal region, after excluding airways, vessels, and pleura. The two radiologists working in consensus chose 100 ROIs for each class, resulting in a total of 600 ROIs. Figure 2 shows 10 ROIs of each class of DILD pattern in HRCT.

In addition to the ROIs, the two radiologists also created whole lung segmentation masks labeling each of the six image pattern classes on 92 randomly selected 2D HRCT images from the DILD patients. These hand-labeled gold-standard data were used to evaluate the results of the deep learning with and without the Perlin noise-based data augmentation methods, and were not used for training.

### Data augmentation using Perlin noise

In this study, we adopted a novel data augmentation technique using Perlin noise to generate a random mixture of class-labelled ROI patches. Perlin noise is a type of gradient noise, and is generally used to generate a natural-appearing pattern in computer graphics and games^5^. A further subtype is improved Perlin simplex noise, which uses a simpler space-filling grid, has fewer directional artifacts, and has a lower computational overhead than the original Perlin noise^6^. In this study, we implemented the python package *noise* (version 1.2.2) to generate 2D Perlin simplex noise. Hereafter, we mean this simplex noise when we mention Perlin noise.

The Perlin noise algorithm first generates a pseudorandom gradient vector on each corner on a given grid. Next, it calculates the distance vectors from a given position to the surrounding corners on the grid. Then, it takes the dot product between the distance vector and the gradient vector, thereby obtaining influence values. The dot product becomes positive if the two vectors are pointing in the same direction, while it becomes negative if the two vectors are pointing in opposite directions. The final step is to interpolate between these influence values, to construct smooth patterns within the grid.

In our experiments, we first generated 2D Perlin noise with the same size of ROIs (20 × 20 pixels). Then we made two subdivided areas; one random region with positive pixel values, and another region with negative pixel values. These two areas can be used as random masks for the ROI augmentation. We choose two randomly selected ROI patches from the six classes and applied the Perlin-generated masks on them, respectively. As a result, we constructed an augmented ROI (size of 20 × 20 pixels) with a combination of two different ROIs with Perlin-generated masks. Although we had only a limited number of ROI patches (600 ROIs in total), we could theoretically generate an infinite number of ROI patches using this method.

### Convolutional neural network

We used a CNN to classify each ROI into one of six classes of DILD. Various CNN models have been applied to computer vision problems such as image classification and segmentation, and they have shown remarkable accuracy in such tasks^3^. In this study, we utilized FusionNet architecture, which is a state-of-the-art network for image segmentation^12^. The network consists of an encoding (downscaling) path to retrieve the features of ROIs, and a symmetric decoding (upscaling) path that enables a prediction to be made on the ROIs. Each path consists of multiple blocks with a combination of different layers, e.g., convolutional, residual, and max-pooling layers, and skip connections between the encoding/decoding paths.

**Figure 3:**
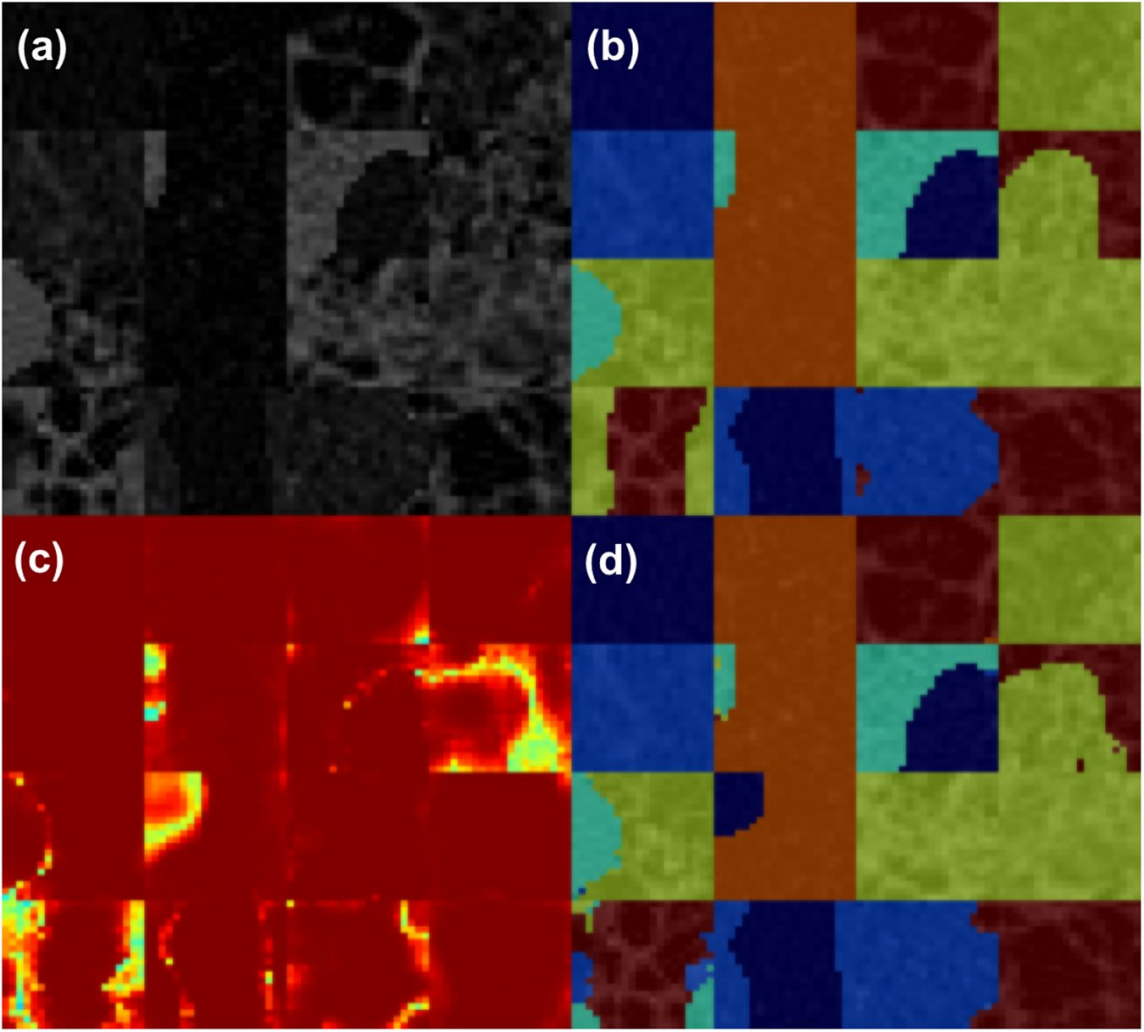
Examples of 10 ROIs (size of 20 × 20 pixel) of each class of DILD image patterns in HRCT. Examples of the CNN training process: (**a**) a batch of 16 ROIs generated using Perlin noise, (**b**) corresponding masks representing the DILD classes for the batch; normal (dark blue), GGO (light blue), consolidation (cyan), RO (yellow), emphysema (orange), and honeycombing (brown), (**c**) softmax probability maps for the batch; high probability (red), low probability (green), and (**d**) predicted labels; color scheme is the same as (**b**). We note that the figure is best viewed in color.

To evaluate the utility of Perlin noise, we performed two experiments with the same ROIs and CNN architecture, but with different data augmentation techniques. First, we adopted conventional data augmentation for the ROIs. We randomly flipped the ROIs up and down, and left and right, and then added zero-mean Gaussian random noise with 1/10 of the standard deviation of the original ROI. Second, we applied our newly developed data augmentation technique using Perlin noise. As for the first experiment, we randomly flipped the ROIs up and down, and left and right, then applied Perlin noise to make ROIs with random masks, instead of adding the random Gaussian noise. We used the same 4 × 4 filter for the convolutional layers. We divided the total 600 ROIs into a training (80%) and test/validation set (20%) for each class. In addition, we adopted a general early stopping strategy to prevent the decrease of training accuracy in validation set due to overfitting^13^. Figure 3 shows an example of the CNN training process with data augmentation using Perlin noise. Note that we did not apply any data augmentation techniques to the test set. For the CNN training, we utilized a NVIDIA Tesla P40 GPU installed on Kakao Brain clouds, and the training took ~1 hour for 10 epochs of 10 000 steps with a batch size of 16.

### Statistical analysis

Paired *t*-tests were used to detect any statistical differences in the classification accuracies between CNNs with data augmentation with and without Perlin noise for the 120 test ROIs. The accuracy of a classifier Γ was defined as follows:

$${\rm{Accuracy}}=\frac{{\rm{1}}}{{N}_{x}{N}_{y}{N}_{ROI}}\sum _{i=1}^{{N}_{x}}\sum _{j=1}^{{N}_{y}}\sum _{k=1}^{{N}_{{ROI}}}\Gamma ({R}_{ijk})\times 100\,( \% )$$

where

$${\rm{\Gamma }}({R}_{ijk})\,=\,\{\begin{array}{l}1,\,if\,correctly\,classifies\,{R}_{ijk}\,into\,one\,of\,the\,six\,classes\\ 0,\,otherwise,\,\end{array}$$

here, *R*_*ijk*_ is the *i* × *j* pixel in the *k*-th ROI of the test data, and *N*_*x*_, *N*_*y*_, and *N*_*ROI*_ are the number of pixels in the x- and y-axis and the number of ROIs, respectively. The classification accuracy would increase if the classifier Γ correctly classified each pixel of the ROIs into one of the six classes, i.e., normal, ground-glass opacity (GGO), consolidation, reticular opacity (RO), emphysema, and honeycombing. In addition, the pixel-by-pixel classification accuracy with both methods of data augmentation was compared for 92 whole lung HRCT images, with a significance level of p = 0.05 being considered to indicate a significant difference.

## Results

The pixel-by-pixel classification accuracies for each DILD class of the 120 ROIs of the test set, both with and without the use of Perlin noise, are shown in Table 1. Overall, the CNN with Perlin noise (89.5%) data augmentation showed a significantly higher accuracy (p < 0.001) than the CNN with conventional data augmentation (82.1%). The CNN with Perlin noise showed a higher accuracy in classifications of normal, GGO, RO, and emphysema classes than did the conventional CNN, although the conventional CNN showed a non-significant slightly higher accuracy for consolidation and honeycombing classes.

**Table 1:** Comparison of pixel-by-pixel classification accuracies between the CNN with and without Perlin noise for 120 test ROIs. CNN-P, CNN with Perlin noise data augmentation; CNN-C, CNN with conventional data augmentation.

Class of image patterns (# of ROIs)	CNN-P (%)	CNN-C (%)	P-value
Normal (20)	98.9	97.7	0.18
GGO (20)	90.0	85.8	0.16
Consolidation (20)	91.6	93.1	0.47
RO (20)	84.3	50.0	<0.001
Emphysema (20)	90.5	83.8	0.12
Honeycombing (20)	81.8	82.3	0.87
Mean	89.5	82.1	<0.001

**Figure 4:**
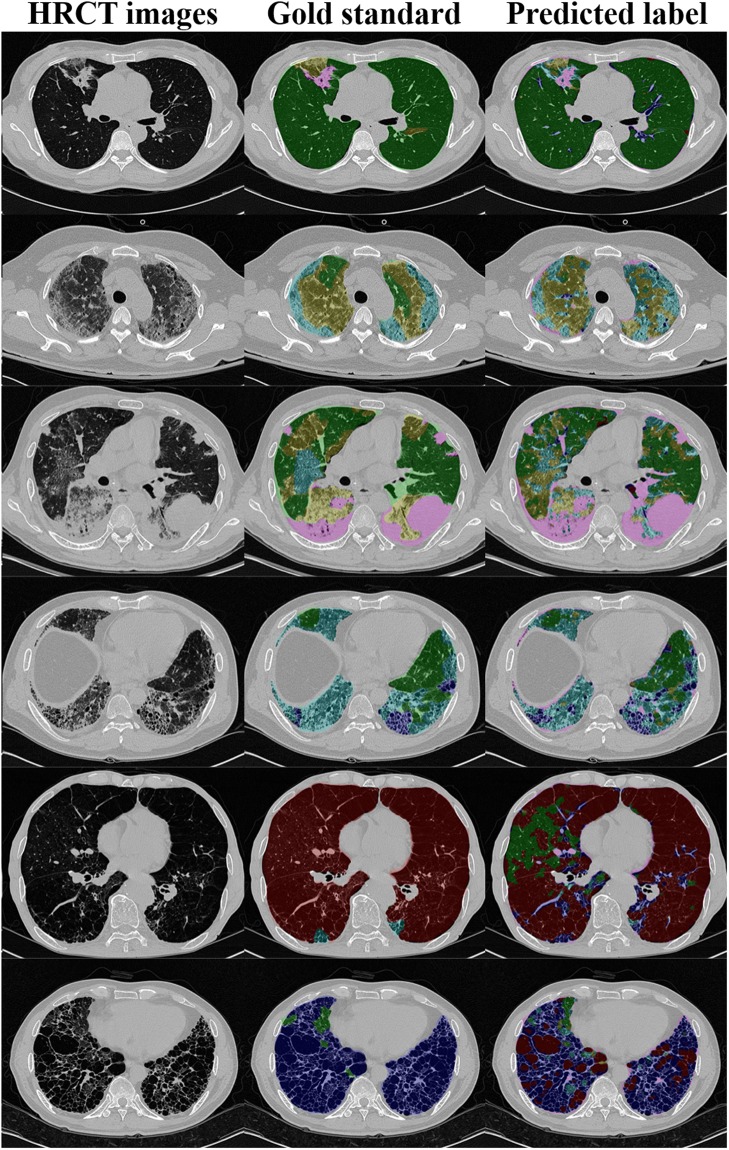
Examples of 2D HRCT images (left) with the corresponding gold-standard classifications of radiologist 2 (middle) and the CNN predicted labels (right). From top to bottom, the dominant patterns of DILD are normal (green), GGO (yellow), consolidation (pink), RO (cyan), emphysema (brown), and honeycombing (blue).

We also compared the image quantification accuracies for 92 whole lung HRCT images, as summarized in Table 2. As there were two different sets of hand-labeled segmentations from the two radiologists, we compared the results individually for each radiologist’s segmentations. As a result, the quantification accuracies for the CNN with Perlin noise were significantly higher (49.8–55.0%, p < 0.001) than those for the conventional CNN (45.7–49.9%). Individually, all classes except honeycombing showed significantly higher accuracies for the CNN with Perlin noise than for the conventional CNN. Only the honeycombing class showed significantly lower accuracy for the CNN with Perlin noise (57.7–63.7%, p < 0.001) than for the conventional CNN (67.6–71.6%). Figure 4 shows examples of the whole lung quantification from the CNN with Perlin noise.

**Table 2:** Comparison of image quantification accuracies of 92 whole lung HRCT images between the CNNs with and without Perlin noise and the radiologists.

DILD class	Inter-radiologist agreement(%)	Radiologist 1			Radiologist 2		
		CNN-P (%)	CNN-C (%)	P-value	CNN-P (%)	CNN-C (%)	P-value
Normal	62.7–92.2	**40**.**2**	36.1	<0.001	**47**.**6**	42.9	<0.001
GGO	15.7–67.5	**38**.**9**	33.8	<0.001	**29**.**4**	23.0	<0.001
Consolidation	52.3–60.4	**48**.**6**	38.5	<0.001	**59**.**1**	48.2	<0.001
RO	45.7–56.0	**67**.**3**	62.2	<0.001	**67**.**8**	61.5	<0.001
Emphysema	18.2–70.0	**71**.**0**	57.2	0.005	**37**.**2**	31.0	0.69
Honeycombing	39.5–82.2	63.7	**71**.**6**	<0.001	57.7	**67**.**6**	<0.001
Mean	45.3–65.1	**55**.**0**	49.9	<0.001	**49**.**8**	45.7	<0.001

For comparison, we evaluated the inter-observer agreements of the radiologists for each class, and found that the radiologists showed the highest agreement for normal patterns (62.7–92.2%), and the lowest agreement with a large variance for GGO patterns (15.7–67.5%). We note that their diagnoses were performed solely independently and were compared the results reaching a consensus.

## Discussion

Compared with the conventional CNN, the CNN with Perlin noise showed a marginally higher ROI-based pixel-by-pixel classification accuracy, and significantly higher whole lung quantification accuracy. There are several possible reasons for the performance improvements with the use of Perlin noise. One is that the data augmentation using Perlin noise seems to effectively prevent overfitting in the deep learning training. When a dataset contains only a small number of cases, as is very common in medical studies, overfitting and its potential to reduce the performance of the deep learning is always an issue. In the present study, the CNN with conventional data augmentation showed unstable changes of loss/accuracy for the validation set while training, and we therefore had to stop the training early. Conversely, the CNN with Perlin noise showed more stable decreases in loss of accuracy for the validation set. Another possible reason is that Perlin noise-generated ROIs seem to provide more diverse combinations of DILD disease patterns. As DILD disease patterns usually show a mixture of several classes within an image, the Perlin noise-generated ROIs could be considered to be more similar to real cases than the ROIs of a single class. Such diverse training datasets increase the robustness of the deep network in comparison with ordinary/simple datasets. Hence, we suggest using a diverse set of training data for deep learning studies, especially with small datasets.

While the use of Perlin noise successfully improved the CNN performance, this method is still subject to several limitations. First, the whole lung quantification accuracies were much lower than the ROI-based classification accuracies. One of the main reasons for this disagreement is that the CNN was trained using ROIs representing typical disease patterns, to allow effective discrimination of the different patterns. However, as can be seen on the clinical HRCT images of the DILD patients, many lung areas are not clearly represented by a typical disease pattern. For example, there may be transitional zones between disease patterns as disease progresses from one state to another. Therefore, there is no clear threshold allowing the discrimination of such borderline patterns. To increase the accuracy of whole lung quantification, more diverse patterns are required for the CNN training, including transitional zones.

Second, the agreement between the CNN results and human radiologists (49.8–55.0%) is within the range, but not exceeding that, of the individual human radiologists (45.3–65.1%). One possible reason for the moderate accuracy in whole lung quantifications is the ambiguous nature of the DILD disease patterns. For example, GGO and RO are highly ambiguous because there is no clear threshold to differentiate between GGO and RO patterns. Hence, there may be a large disagreement in the classification of these patterns, even among radiologists. Another possible reason is the different quantification methods applied between the CNN and human. While the CNN assessed lung parenchyma on a pixel-by-pixel basis, the human radiologists were asked to draw lines discriminating different disease patterns on a region-by-region basis. In this case, small areas of local disease patterns or normal vessels within the lung parenchyma could be easily ignored.

Third, there is possibly a data clustering issue as we chose several ROIs from a single patient. Although we tried to select one ROI from each slice to minimize any clustering effect, it is possible that we may not have had sufficiently diverse patterns for each class. Fourth, the CNN with Perlin noise showed significantly lower accuracies for honeycombing patterns than did the conventional CNN. A honeycombing pattern is defined as a honeycomb-like feature with surrounding walls. It is possible that the Perlin noise-generated ROIs were not able to represent the typical patterns of honeycombing, as representative patterns larger than the Perlin noise-generated masks could have been destroyed. Hence, for CNN training, it is wise to use Perlin noise appropriate for the properties of the target patterns, especially size.

Last, we only used 2D HRCT images and masks for DILD patterns, because imaging protocol of HRCT is mainly 2D images acquisition including image series with 1 mm slice thickness and 9 mm intervals, which is a historical CT protocol for chest radiology and very important for survival study. We believe that our Perlin noise-based augmentation strategy can be also applicable to 3D masks and will improve the performance of classification and quantification. In our future work, we plan to use 3D volumetric images and masks for DILD in order to investigate the effect of different dimensionalities for CNN training and inference as well as the effect of Perlin noise-based augmentation strategy.

## Conclusion

We developed a novel data augmentation strategy using Perlin noise and applied it to the CNN-based automatic classification of DILD disease patterns from 2D HRCT images. In the differentiation of the six typical disease patterns of DILD, the CNN with Perlin noise provided higher accuracy than the CNN with conventional augmentation methods. The results demonstrate both the importance of diverse datasets for deep learning training, and the utility of Perlin noise for helping to create such diversity, which is especially important in cases with limited datasets. Hence, we propose the Perlin noise-based data augmentation strategy to increase the performance of CNN models for classifying and quantifying not only different disease patterns but also general image textures.
